# Supplementary material for: Broadband angular spectrum differentiation using dielectric metasurfaces
Source: Nat Commun. 2024 Mar 12;15:2237. doi: 10.1038/s41467-024-46537-9 (PMC10933489; doi:10.1038/s41467-024-46537-9)
Supplement: Supplementary file 1 — Supplementary Information [file 41467_2024_46537_MOESM1_ESM.pdf]

Supplementary Information for

Broadband angular spectrum differentiation using dielectric metasurfaces

Ming Deng[1,†], Michele Cotrufo[2,3,†], Jian Wang[1], Jianji Dong[1], Zhichao Ruan[4], Andrea Alù[2,*] & Lin Chen[1,5,*]

[1] Wuhan National Laboratory for Optoelectronics and School of Optical and Electronic Information, Huazhong University of Science and Technology, Wuhan 430074, China

[2] Photonics Initiative, Advanced Science Research Center, City University of New York, New York, NY 10031, USA

[3] The Institute of Optics, University of Rochester, Rochester, New York 14627, USA

[4] School of Physics, Zhejiang Province Key Laboratory of Quantum Technology and Device, and State Key Laboratory for Extreme Photonics and Instrumentation, Zhejiang University, Hangzhou 310027, China

[5] Shenzhen Huazhong University of Science and Technology Research Institute, Shenzhen 518063, China

* Corresponding author's email: aalu@gc.cuny.edu; chen.lin@mail.hust.edu.cn

† These authors contributed equally.

Contents

Supplementary Note 1: l, C_j and (m_j, n_j) for three types of angular spectrum differentiation

Supplementary Note 2: Derivation of Eq. (3), $\theta(x, y)$ for the other two types of meta-differentiators and optimization of the cross-sectional geometry

Supplementary Note 3: Image resolution in the Fourier domain

Supplementary Note 4: Output angular spectrum intensity distributions at some discrete wavelengths within 450 nm and 1000 nm

Supplementary Note 5: Bandwidth limitations for realizing high order meta-differentiators

Supplementary Note 6: Theoretical output field intensity distributions

Supplementary Note 7: Theoretical model of the experimental setup for extracting angular spectrum differentiation

Supplementary Note 8: Derivation of angular spectrum differentiation for three parallel rectangular holes

Supplementary Note 9: Angular spectrum differentiation of a single circular hole

Supplementary Note 10: Derivation of transmitted electric field when a meta-differentiator is involved in a 4F system

Supplementary Note 11: Transmission efficiency of the cross-polarized transmitted field

Supplementary Note 1: l, C_j and (m_j, n_j) for three types of angular spectrum differentiation

Supplementary Table S1 | l, C_j and (m_j, n_j) for three types of angular spectrum differentiation

l	$l = 1$		$l = 2$
C_j	C_1	C_1	$C_1 = C_2$
(m_j, n_j)	$(m_1, n_1) = (1, 1)$	$(m_1, n_1) = (1, 0)$	$(m_1, n_1) = (1, 0)$,<br>$(m_2, n_2) = (0, 1)$
$t(x, y)$	$-C_1xy$	$-\mathrm{i}C_1x$	$-\mathrm{i}C_1(x + y)$
$\hat{H}$	$C_1\frac{\partial^2}{\partial k_x \partial k_y}$	$C_1\frac{\partial}{\partial k_x}$	$C_1(\frac{\partial}{\partial k_x}+\frac{\partial}{\partial k_y})$

Supplementary Note 2: Derivation of Eq. (3), $\theta(x, y)$ for the other two types of meta-differentiators and optimization of the cross-sectional geometry

As shown in Fig. 1d in the main text, the nanopillar is rotated with an orientation angle θ anticlockwise. The local transmission Jones matrix can be expressed as $\mathbf{T}(\theta = 0) = \begin{bmatrix} t_u & 0 \\ 0 & t_v \end{bmatrix}$ with $\theta = 0$. When $\theta \neq 0$, the local transmission Jones matrix is[1]

$$\mathbf{T}(\theta) = \mathbf{S}(-\theta)\mathbf{T}(\theta = 0)\mathbf{S}(\theta) = \begin{bmatrix} t_u\cos^2\theta + t_v\sin^2\theta & \frac{t_u - t_v}{2}\sin 2\theta \\ \frac{t_u - t_v}{2}\sin 2\theta & t_u\sin^2\theta + t_v\cos^2\theta \end{bmatrix} \tag{S1}$$

where $\mathbf{S}(\theta) = \begin{bmatrix} \cos\theta & \sin\theta \\ -\sin\theta & \cos\theta \end{bmatrix}$ is the rotation matrix. The two off-diagonal elements are the same with $\sigma = \frac{t_u - t_v}{2}\sin 2\theta$, indicating that the cross-polarized transmission coefficients under x- or y-polarized incidence is the same.

For the second type with $t(x, y) = -\mathrm{i}C_1x$, θ should satisfy $\sin 2\theta = -\mathrm{i}\frac{2C_1}{t_u - t_v}x$ with $x \in [-a/2, a/2]$ and $y \in [-a/2, a/2]$. $|C_1| \leq \left|\mathrm{i}\frac{t_u - t_v}{a}\right|$ holds with $x = \pm a/2$ since $|\sin 2\theta| \leq 1$. In order to maximize the efficiency of the output angular spectrum, C_1 should be chosen to be $\pm\mathrm{i}\frac{t_u - t_v}{a}$, associated with $\theta = \pm\arcsin(2x / a) / 2$. For the third type with $t(x, y) = -\mathrm{i}C_1(x + y)$, θ should satisfy $\sin 2\theta = -\mathrm{i}\frac{2C_1}{t_u - t_v}(x + y)$ with $x \in [-a/2, a/2]$ and $y \in [-a/2, a/2]$. $|C_1| \leq \left|\mathrm{i}\frac{t_u - t_v}{2a}\right|$ holds with $(x, y) = (-a/2, -a/2)$ or $(a/2, a/2)$ since $|\sin 2\theta| \leq 1$. C_1 should be chosen to be $\pm\mathrm{i}\frac{t_u - t_v}{2a}$, so as to maximize the efficiency of the output angular spectrum, corresponding to $\theta = \pm\arcsin[(x + y) / a)] / 2$.

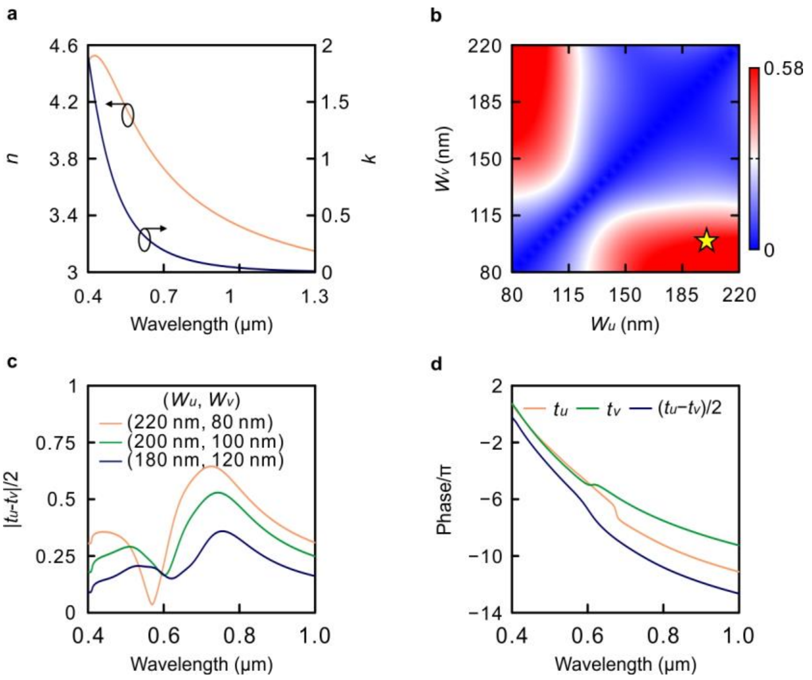

Supplementary Figure S1. Optimization of the nanopillar's cross-sectional geometry. a Refractive index and extinction coefficient of silicon measured by an ellipsometer versus wavelength. **b** Amplitude of $\frac{t_u - t_v}{2}$ versus W_u and W_v at 685.5 nm. **c** Amplitude of $\frac{t_u - t_v}{2}$ versus wavelength with three groups of cross-sectional parameters. **d** Phase of t_u, t_v and $\frac{t_u - t_v}{2}$ versus wavelength with $W_u = 200$ nm and $W_v = 100$ nm.

The refractive index and extinction coefficient of silicon layer fabricated by magnetron sputtering was measured by an ellipsometer (Supplementary Fig. S1a). The efficiency and the phase delay for the output angular spectrum are affected by $\frac{t_u - t_v}{2}$. To obtain the large efficiency at 450, 532 and 685.5 nm, we have firstly simulated the amplitude of $\frac{t_u - t_v}{2}$ versus W_u and W_v, when h and P are fixed at 220 nm and 280 nm, respectively (Supplementary Fig. S1b). We found the cross-sectional parameters of W_u = 200 nm and W_v = 100 nm can make the amplitude of $\frac{t_u - t_v}{2}$ approach the maximum value. The geometrical parameters with a slight deviation from W_u = 200 nm and W_v = 100 nm might further increase the amplitude at 685.5 nm (Supplementary Fig. S1c), but lead to fabrication complexity since less nanopillar width or gap separation is used. We have used W_u = 200 nm and W_v = 100 nm for the design and experimental demonstration of the three meta-differentiators for ease of fabrication. The amplitude dip around 600 nm is attributed to the equal phase of t_u and t_v (Supplementary Fig. S1d).

Supplementary Note 3: Image resolution in the Fourier domain

We here investigate the image resolution for these three types of meta-differentiator, when the input Gaussian beam has a waist radius, w, and field profile of $E_p(x, y) = \exp[-(x^2 + y^2) / w^2]$. For the first type $t(x,y)=\begin{cases}-C_1xy & (|x|,|y|\le a/2)\\ 0 & \text{otherwise}\end{cases}$, the angular spectrum of the output field is $A_p(k_x, k_y) = -C_1\left[\int_{-a/2}^{a/2} x\exp(-x^2/\mathrm{w}^2)\exp(-\mathrm{i}k_x x)\mathrm{d}x\right]\left[\int_{-a/2}^{a/2} y\exp(-y^2/w^2)\exp(-\mathrm{i}k_y y)\mathrm{d}y\right]$. With the definition $F(n, k_x, w, a) = \int_{-a/2}^{a/2} x^n\exp(-x^2/\mathrm{w}^2)\exp(-\mathrm{i}k_x x)\mathrm{d}x$, we have

$$A_p(k_x, k_y) = -C_1F(1, k_x, w, a)F(1, k_y, w, a) \tag{S2}$$

With $F(0, k_x, w, a) = 2\int_0^{a/2}\exp(-x^2/\mathrm{w}^2)\cos(k_x x)\mathrm{d}x$, we can obtain

$$\frac{\partial F(0, k_x, w, a)}{\partial k_x} = w^2\left[\exp\left(-\frac{a^2}{4w^2}\right)\sin\left(\frac{k_x a}{2}\right)-\frac{1}{2}k_xF(0, k_x, w, a)\right] \tag{S3}$$

And

$$\frac{\partial\left[\exp\left(k_x^2w^2/4\right)F(0, k_x, w, a)\right]}{\partial k_x} = \exp\left(\frac{k_x^2w^2}{4}\right)\left[\frac{1}{2}k_xw^2F(0, k_x, w, a)+\frac{\partial F(0, k_x, w, a)}{\partial k_x}\right] \tag{S4}$$

By combing Supplementary Eq. (S3) and Supplementary Eq. (S4), we can get $F(0, k_x, w, a) = \exp\left(-k_x^2w^2/4\right)w^2\exp(-0.25a^2/w^2)\int\exp\left(k_x^2w^2/4\right)\sin(0.5k_xa)\mathrm{d}k_x$, which is further transferred to $F(0, k_x, w, a) = \frac{w^2\exp\left(-k_x^2w^2/4\right)}{2\mathrm{i}}\int\left\{\exp\left[\left(\frac{k_xw}{2}+\frac{\mathrm{i}a}{2w}\right)^2\right]-\exp\left[\left(\frac{k_xw}{2}-\frac{\mathrm{i}a}{2w}\right)^2\right]\right\}\mathrm{d}k_x$. The final expression of $F(0, k_x, w, a)$ can be written as

$$F(0, k_x, w, a) = \frac{\sqrt{\pi}w\exp\left(-k_x^2w^2/4\right)}{2\mathrm{i}}\left[\mathrm{erfi}\left(\frac{k_xw}{2}+\frac{\mathrm{i}a}{2w}\right)-\mathrm{erfi}\left(\frac{k_xw}{2}-\frac{\mathrm{i}a}{2w}\right)\right], \tag{S5}$$

where $\mathrm{erfi}(x)$ is the imaginary error function, defined as $\mathrm{erfi}(x)=\frac{2}{\sqrt{\pi}}\int_0^x\exp(t^2)\mathrm{d}t$. Through the relationship between $F(1, k_x, w, a)$ and $F(0, k_x, w, a)$, $F(1, k_x, w, a)$ can thus be expressed as $F(1, k_x, w, a) = -2\mathrm{i}\int_0^{a/2} x\exp(-x^2/w^2)\sin(k_x x)\mathrm{d}x = \mathrm{i}\frac{\partial F(0, k_x, w, a)}{\partial k_x}$, which is finally transformed to

$$F(1, k_x, w, a) = \mathrm{i}w^2\exp\left(-\frac{a^2}{4w^2}\right)\sin\left(\frac{k_xa}{2}\right)-\frac{\sqrt{\pi}k_xw^3}{4}\exp\left(-\frac{k_x^2w^2}{4}\right)\left[\mathrm{erfi}\left(\frac{k_xw}{2}+\frac{\mathrm{i}a}{2w}\right)-\mathrm{erfi}\left(\frac{k_xw}{2}-\frac{\mathrm{i}a}{2w}\right)\right] \tag{S6}$$

By incorporating Supplementary Eq. (S6) into Supplementary Eq. (S2), we can finally obtain $A_p(k_x, k_y)$. With a = 300 μm and w = 400 μm, the normalized $|A_p(k_x, k_y)|^2/|A_p(k_x, k_y)|^2_{\max}$ is shown in Supplementary Fig. S2a. There are many side lobes around the four main peaks next to the center of angular spectrum domain, where the maximum side lobe is bounded by the green dashed circle. The intensity ratio of the maximum side lobe to the main peak versus a and w is plotted in Supplementary

Fig. S2b. The image resolution, is defined as the intensity ratio is 0.01, below which the meta-differentiator can well achieve angular spectrum differentiation. The maximum waist radius, w, allowed for the input beam is increased with the enlarged meta-differentiator, which is represented by the green dashed line in Supplementary Fig. S2b. The waist radius of the input beam in the Fourier domain should be larger than $2/w$. With a = 300 μm, the maximum waist radius, w, allowed for the input beam is estimated to be 108 μm, which corresponds to the waist radius in the Fourier domain with $2/w$=18.5 mm^{-1}.

For the second type $t(x,y)=\begin{cases} -iC_1 x & (|x|,|y|\le a/2) \\ 0 & \text{otherwise} \end{cases}$, the angular spectrum of the output field is $A_p(k_x, k_y) = -iC_1F(1, k_x, w, a)F(0, k_y, w, a)$. With a = 300 μm and w = 400 μm, the normalized $|A_p(k_x, k_y)|^2/|A_p(k_x, k_y)|^2_{\max}$ is shown in Supplementary Fig. S2c. The intensity ratio of the maximum side lobe to the main peak versus a and w is plotted in Supplementary Fig. S2d. With the same definition of image resolution, the waist radius of the input beam in the Fourier domain should be larger than $2/w$ as well. With a = 300 μm, the maximum waist radius, w, allowed for the input beam is estimated to be 108 μm, which corresponds to the waist radius in the Fourier domain with $2/w$=18.5 mm^{-1}.

For the third type $t(x,y)=\begin{cases} -iC_1 (x+y) & (|x|,|y|\le a/2) \\ 0 & \text{otherwise} \end{cases}$, the angular spectrum of the output field is $A_p(k_x, k_y) = -iC_1[F(1, k_x, w, a)F(0, k_y, w, a) + F(0, k_x, w, a)F(1, k_y, w, a)]$. With a = 300 μm and w = 400 μm, the normalized $|A_p(k_x, k_y)|^2/|A_p(k_x, k_y)|^2_{\max}$ is shown in Supplementary Fig. S2e. The intensity ratio of the maximum side lobe to the main peak versus a and w is plotted in Supplementary Fig. S2f. With the same definition of image resolution, the waist radius of the input beam in the Fourier domain should be larger than $2/w$ as well. With a = 300 μm, the maximum waist radius, w, allowed for the input beam, is estimated to be 116 μm, which corresponds to the waist radius in the Fourier domain with $2/w$=17.2 mm^{-1}.

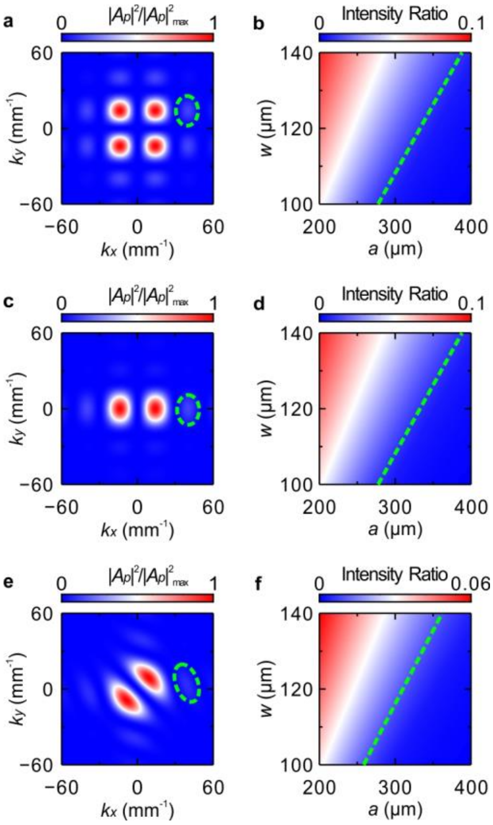

Supplementary Figure S2. Image resolution. a, c, e Normalized $|A_p(k_x, k_y)|^2/|A_p(k_x, k_y)|^2_{\max}$ with a = 300 µm and w = 400 µm for the first (**a**), second (**c**) and third types (**e**). One of the maximum side lobes is bounded by the green dashed circles. **b, d, f** Intensity ratio of the maximum side lobe to the main peak for the first (**b**), second (**d**) and third types (**f**), where the green dashed lines indicate the intensity ratio is 0.01.

Supplementary Note 4: Output angular spectrum intensity distributions at some discrete wavelengths within 450 nm and 1000 nm

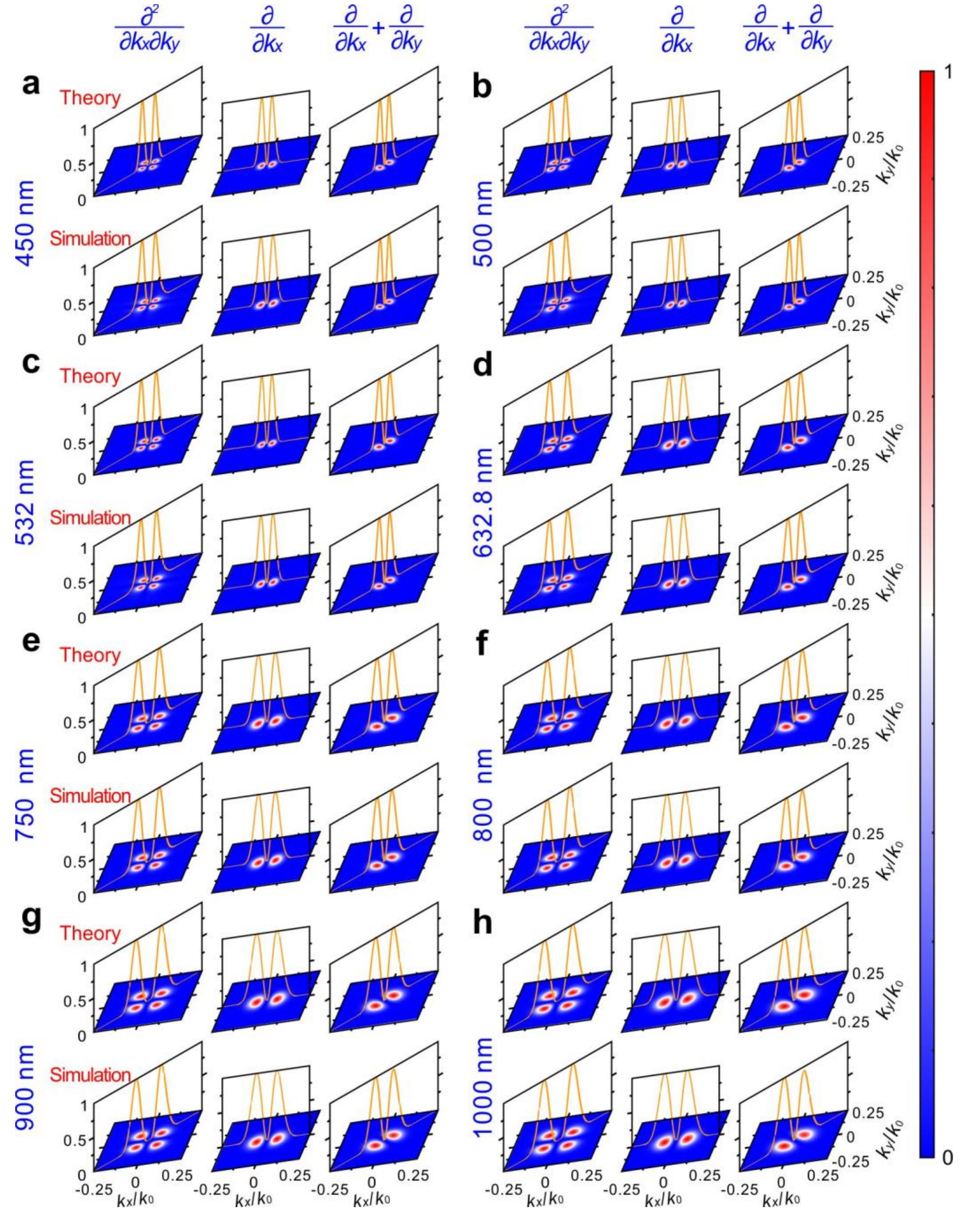

Supplementary Figure S3. Output angular spectrum intensity distributions for the three types of differentiations: 450 (a), 500 (b), 532 (c), 632.8 (d), 750 (e), 800 (f), 900 (g) and 1000 nm (h).

Supplementary Note 5: Bandwidth limitations for realizing high order meta-differentiators

The proposed method can be extended to design arbitrary order angular spectrum meta-differentiators in principle. However, the working bandwidth is limited as differentiation order is increased. Here, we have designed three other meta-differentiators composed of 60×60 nanopillars with $\hat{H} \propto \frac{\partial^2}{(\partial k_x)^2}$, $\frac{\partial^3}{(\partial k_x)^3}$ and $\frac{\partial^4}{(\partial k_x)^4}$. By using the same method described in Supplementary Note 2, $\theta = \arcsin\left[\frac{x^2}{(8.26\ \mu\text{m})^2}\right]/2$, $\theta = \arcsin\left[\frac{x^3}{(8.26\ \mu\text{m})^3}\right]/2$ and $\theta = \arcsin\left[\frac{x^4}{(8.26\ \mu\text{m})^4}\right]/2$ are derived, respectively. The corresponding l, C_j and (m_j, n_j) for the three types of angular spectrum differentiation are listed in Supplementary Table S2 and the metasurface distributions are shown in Supplementary Fig. S4a. The angular spectrum distributions of $|\hat{H}A_x|^2/|\hat{H}A_x|^2_{\text{max}}$ with theoretical calculation and $|A_y|^2/|A_y|^2_{\text{max}}$ with FDTD simulation are shown in Supplementary Figs. S4b-g. The angular spectrum intensity distributions are split into three, four and five parts along k_x direction with the three meta-differentiators, respectively. We can see well consistency between the theoretical and the simulation results in the wavelength range around 685.5 nm, but the deviation between theory and simulation becomes clearer as the working wavelength is far from the central wavelength, especially for high order differentiation.

Supplementary Table S2 | l, C_j and (m_j, n_j) for three types of angular spectrum differentiation

l	$l = 1$		
C_j	C_1	C_1	C_1
(m_j, n_j)	$(m_1, n_1) = (2, 0)$	$(m_1, n_1) = (3, 0)$	$(m_1, n_1) = (4, 0)$
$t(x, y)$	$-C_1x^2$	$\mathrm{i}C_1x^3$	C_1x^4
$\hat{H}$	$C_1\frac{\partial^2}{(\partial k_x)^2}$	$C_1\frac{\partial^3}{(\partial k_x)^3}$	$C_1\frac{\partial^4}{(\partial k_x)^4}$

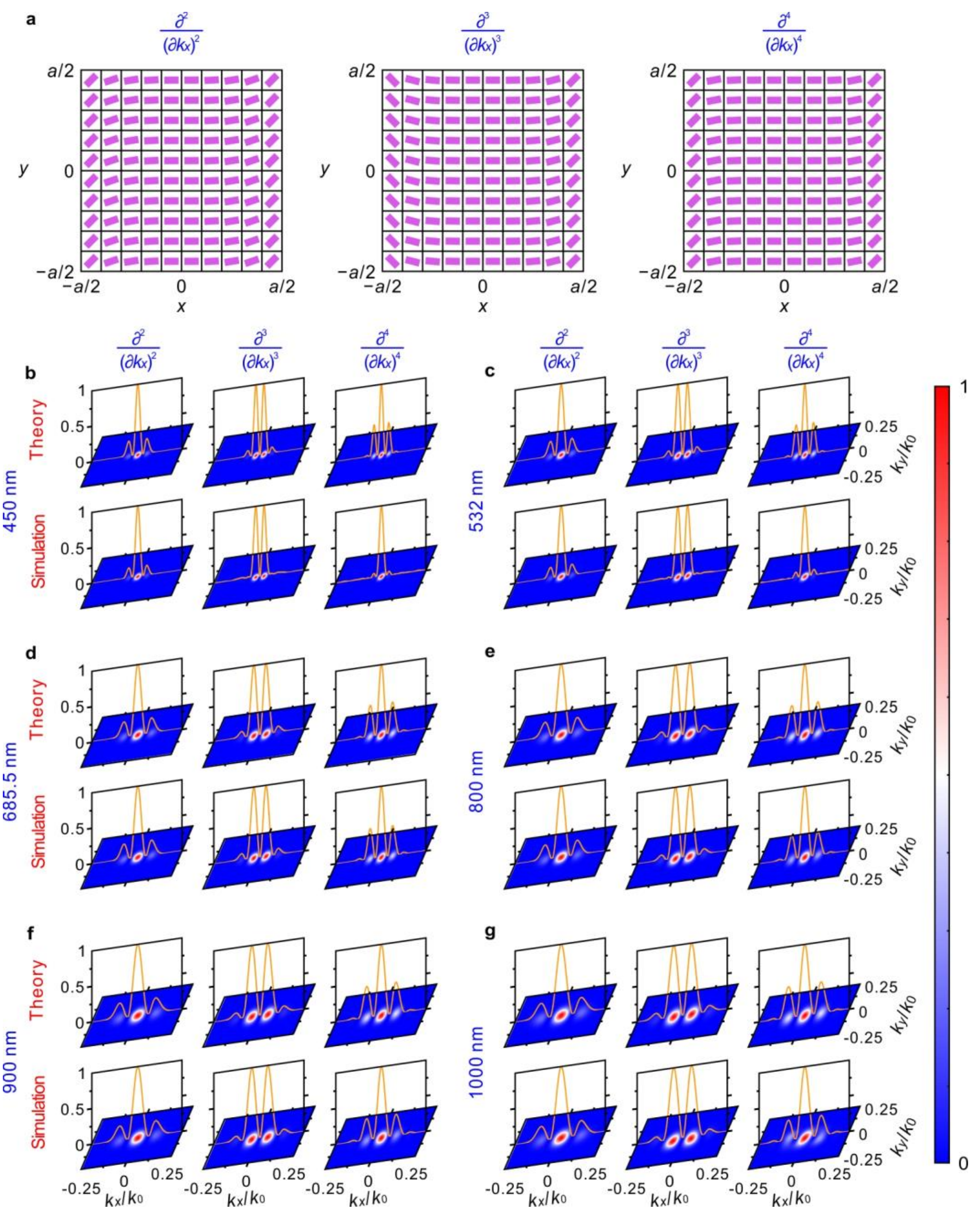

Supplementary Figure S4. High order meta-differentiators. a Schematics of the top view presenting the distribution of the orientation angles of the three meta-differentiators with $\frac{\partial^2}{(\partial k_x)^2}$, $\frac{\partial^3}{(\partial k_x)^3}$ and $\frac{\partial^4}{(\partial k_x)^4}$, respectively. **b-g** Output angular spectrum intensity distributions for the three types of differentiations at 450 (**b**), 532 (**c**), 685.5 (**d**), 800 (**e**), 900 (**f**), and 1000 nm (**g**), under normal incidence of *x*-polarized Gaussian beam.

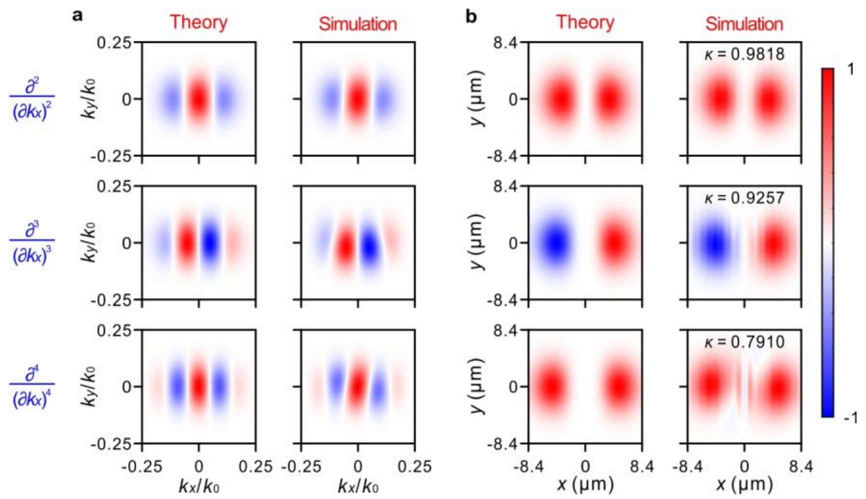

Supplementary Figure S5. Output angular spectrum and field intensity distributions at 1000 nm. **a** Distributions of the real part of angular spectrum electric field at 1000 nm. **b** Distributions of the real part of near field at 1000 nm. For each figure, the electric field is normalized with the complex field, associated with the maximum amplitude.

Based on the theoretical calculation, the output angular spectrum is always symmetric or antisymmetric with respect to k_x and k_y axes simultaneously (Supplementary Fig. S5a). But in the long wavelength range, the simulated results clearly indicate apparent deviation with those from theoretical calculation: the output angular spectrum is not strictly symmetric or antisymmetric with respect to k_x and k_y axes simultaneously. The required near-field distributions for achieving the three types of differentiation should be symmetric or antisymmetric (Supplementary Fig. S5b). However, the near-field intensity distributions offered by the meta-differentiators are more or less different from the theoretical distributions. For the two even order meta-differentiators, E_y is not symmetric with respect to x and y axes due to asymmetrical distributions of meta-differentiators (see Supplementary Fig. S4a). For the one odd order meta-differentiator, E_y is antisymmetric with respect to y axis only, consistent with the used meta-differentiator. In other words, the transmission field provide by metasurfaces at long wavelengths is not consistent with the required values described by the real-space transfer function, $t(x, y) = \sum_{j=1}^{l}\left[C_j(\text{-i}x)^{m_j}(\text{-i}y)^{n_j}\right]$.

The inconsistency between the theory and simulations at long wavelengths comes from the stronger coupling between the adjacent nanopillars, since the nanopillars have weaker localization on light. In this situation, $\sigma = \frac{t_u - t_v}{2}\sin 2\theta$ cannot precisely describe the transmission coefficients provided by the metasurfaces at long wavelengths. This deviation can be quantitatively evaluated by the overlap integral

between the simulated and the theoretical transmitted fields[2], $\kappa = \frac{\left\{\iint_{-\infty}^{+\infty}[t(x,y)E_x]E_y^* \mathrm{d}x\mathrm{d}y\right\}\left\{\iint_{-\infty}^{+\infty}[t(x,y)E_x]^* E_y \mathrm{d}x\mathrm{d}y\right\}}{\left\{\iint_{-\infty}^{+\infty}[t(x,y)E_x][t(x,y)E_x]^* \mathrm{d}x\mathrm{d}y\right\}\left(\iint_{-\infty}^{+\infty} E_y E_y^* \mathrm{d}x\mathrm{d}y\right)}$. The calculated κ for different differentiation orders at 1000 nm is presented in Supplementary Fig. S5b. The deviation becomes more apparent for higher order. Consequently, the designed higher order meta-differentiators have worse performance at long wavelengths. Based on the lattice constant used in our work, P = 280 nm, it might be challenging to achieve better differentiation performance at long wavelengths. An effective method of optimizing the differentiation performance at long wavelengths can be made by redesigning the meta-differentiators using a larger lattice constant. However, the resultant meta-differentiators have better performance at long wavelengths, but which is at the sacrifice of poorer performance at short wavelengths. In other words, it is challenging to optimize the performance at long wavelengths, while keeping the central wavelength fixed.

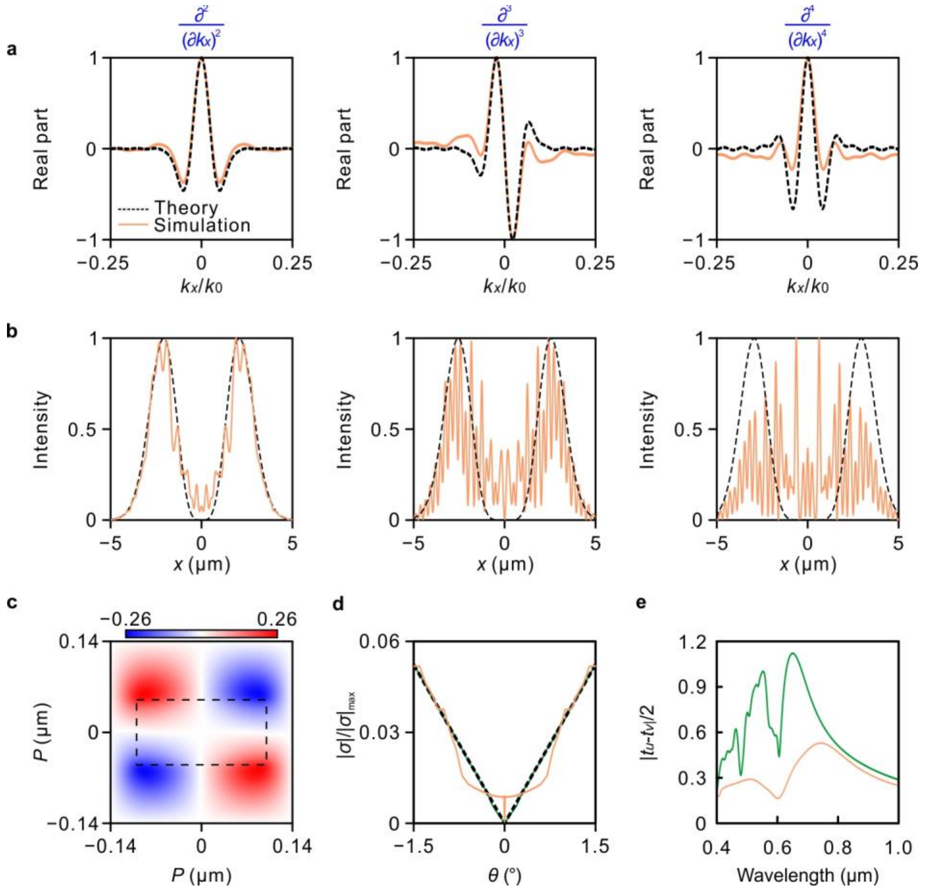

Supplementary Figure S6. Theoretical and simulated deviation for high order differentiation at 450 nm. a Profiles of the real part of angular spectrum electric field at 450 nm along the line $k_y = 0$. The electric field is normalized with the complex field, associated with the maximum amplitude. **b** Corresponding real part of the near-field profiles along the line $y = 0$. **c** Distributions of the real part of near field when a nanopillar unit with $\theta = 0.01°$ is illuminated with x-polarized incidence. The cross section of the nanopillar is outlined by the black dashed lines. **d** $|\sigma|/|\sigma|_{max}$ versus θ as θ is around zero: theory (black dashed line), amorphous silicon nanopillar (yellow line) and crystalline silicon nanopillar (green line). **e** Amplitude of $\frac{t_u - t_v}{2}$ versus wavelength with the crystalline silicon metasurfaces (green line), which shows enhanced transmission efficiency, as opposed to amorphous silicon metasurfaces (yellow line).

In the short wavelength range, we can see apparent deviation between theory and simulation, i.e., the main/side lobe intensity is not consistent near $k_y = 0$, especially for high order differentiation (Supplementary Fig. S6a). This can be attributed to the fact that the corresponding near-field profiles offered by the meta-differentiators deviate from those predicted by theory (Supplementary Fig. S6b). In

our work, we have used the transmission coefficients for E_y under x-polarized incidence, $\sigma = \frac{t_u - t_v}{2}\sin 2\theta$, to design meta-differentiators, but the scattering from the vertices of the nanopillars is neglected (Supplementary Fig. S6c). However, the E_y component, originating from the scattering around the vertices of the nanopillars, is dominant around $\theta = 0$. Consequently, the simulated $|\sigma|$ deviates from the theoretical values when θ is around zero (see Supplementary Fig. S6d). This deviation will lead to the inconsistence of the differentiation field intensity distributions between the theory and simulation (see Supplementary Fig. S6a). For higher order meta-differentiators, there are more nanopillars, associated with small θ, arranged around $x = 0$. As a result, the field intensity distributions offered by the metasurfaces around $x = 0$ show larger deviation for higher order differentiation, as opposed to lower order differentiation (see Supplementary Fig. S6b). This can be used to explain why the designed higher order meta-differentiators have worse performance at short wavelengths.

The feasible method of optimizing the differentiation performance at short wavelengths can be made by using low-loss crystalline silicon nanopillars to construct the meta-differentiators. In this case, we can optimize the crystalline silicon nanopillars to enhance $\left|\frac{t_u - t_v}{2}\right|$, so as to increase $|\sigma|$. As a result, the portion of E_y component originating from the scattering will be reduced. We have used low-loss crystalline silicon nanopillars to optimize the bandwidth of the meta-differentiators. The designed meta-differentiators with the crystalline silicon nanopillar with W_u = 140 nm, W_v = 60 nm, H = 600 nm and P = 280 nm exhibit higher $\left|\frac{t_u - t_v}{2}\right|$ (see Supplementary Fig. S6e). We present in Supplementary Fig. S6d that, the simulated $|\sigma|$ coincides well with the theory, when the crystalline silicon nanopillars are used. The simulated angular spectrum differentiation distributions with the crystalline silicon at 450 nm are well consistent with the theory. In contrast, the simulated angular spectrum differentiation distributions with the amorphous silicon show notable difference with the theory, especially for higher order differentiators (see Supplementary Fig. S7).

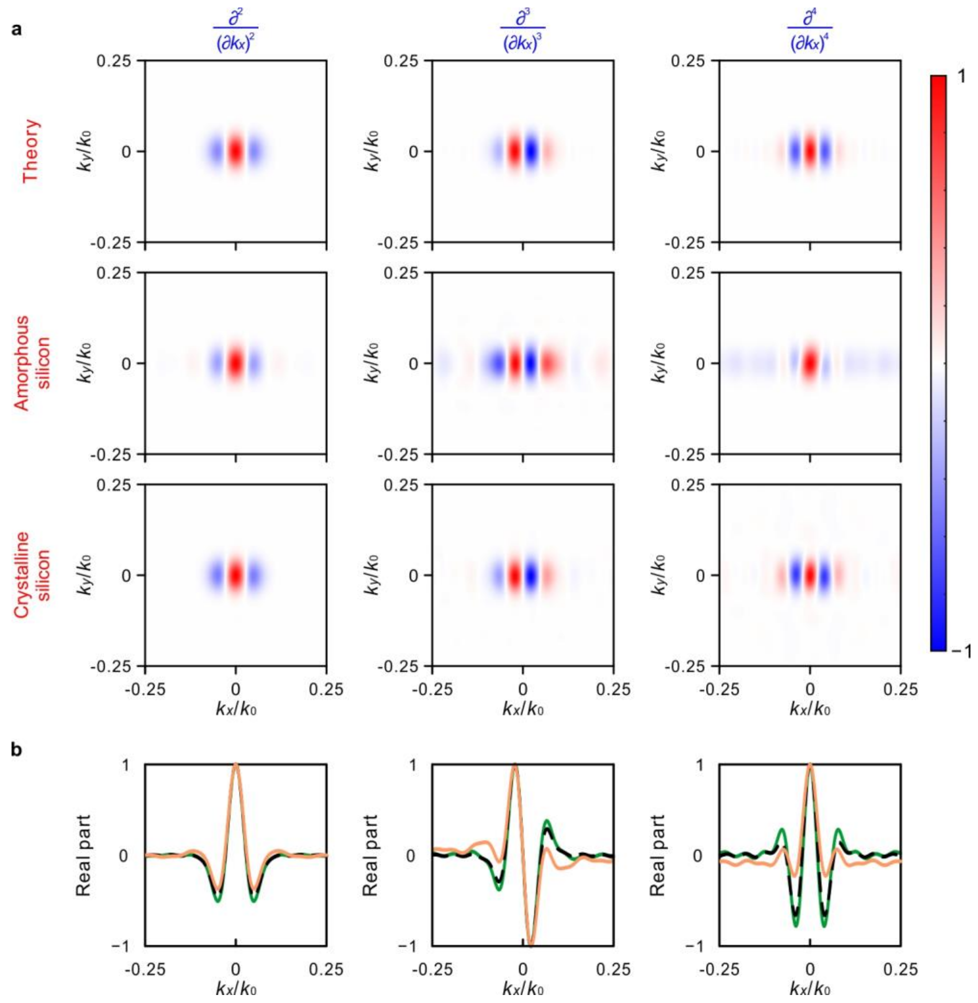

Supplementary Figure S7. Optimization of the bandwidth in the short wavelength range. a Distributions of the real part of the angular spectrum electric field at 450 nm. **b** Profiles of the real part of the angular spectrum electric field along $k_y = 0$ at 450 nm: theory (black dashed line), amorphous silicon nanopillar (yellow line) and crystalline silicon nanopillar (green line).

Supplementary Note 6: Theoretical output field intensity distributions

To check the distribution of the transmission fields of the three types of meta-differentiators, we have evaluated the theoretical results of the three metasurfaces with the same size as those used in Fig. 3 in the main text. The transmission field is retrieved by $|t(x, y)E_x|^2/|t(x, y)E_x|^2_{\max}$.

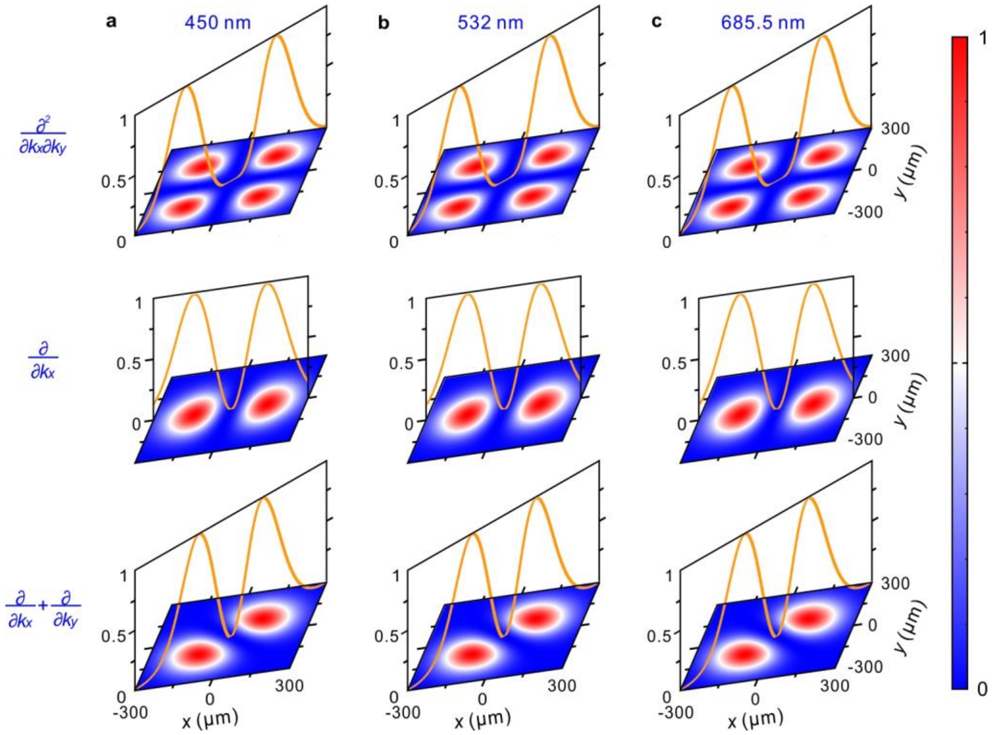

Supplementary Figure S8. Theoretical output field intensity distributions. a-c Output field intensity distributions theoretically retrieved by $|t(x, y)E_x|^2/|t(x, y)E_x|^2_{\max}$ for the three types of meta-differentiators with $\frac{\partial^2}{\partial k_x \partial k_y}$, $\frac{\partial}{\partial k_x}$ and $\frac{\partial}{\partial k_x}+\frac{\partial}{\partial k_y}$ at three wavelengths: 450 nm (**a**), 532 nm (**b**) and 685.5 nm (**c**).

Supplementary Note 7: Theoretical model of the experimental setup for extracting angular spectrum differentiation

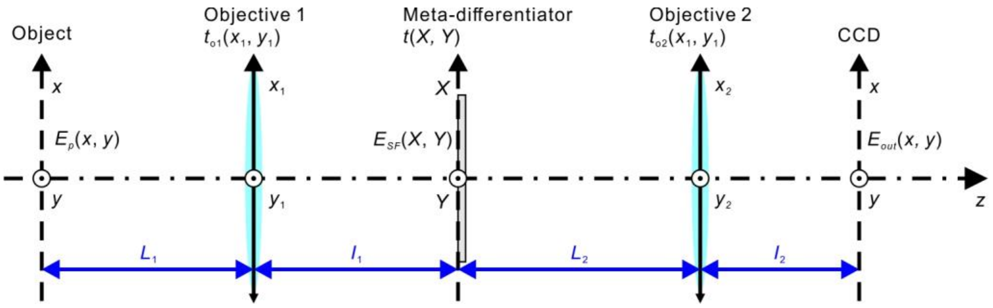

Supplementary Figure S9. Theoretical model for measuring the angular spectrum differentiation. Schematic of the theoretical model for the experimental setup in Fig. 4a in the main text. Here, the polarizers 1 and 2 are removed in this theoretical model, since they are only used to attenuate the co-polarized light beams in the experiment.

The theoretical model for the experimental setup in Fig. 4a is schematically shown in Supplementary Fig. S9, where the complex transmission functions for objectives 1 and 2 are $t_{o1}(x_1, y_1) = \exp\left(-\mathrm{i}k_0\frac{x_1^2+y_1^2}{2F_1}\right)$, $t_{o2}(x_2, y_2) = \exp\left(-\mathrm{i}k_0\frac{x_2^2+y_2^2}{2F_2}\right)$, and F_1, F_2 are the focal lengths, respectively. Considering an input field at a distance of L_1 in front of objective 1, $E_p(x, y)$, the electric field $E_{SF}(X, Y)$, at a distance I_1 after the objective 1 can be marked by the function $\psi(E_p(x, y), X, Y, L_1, I_1, F_1)$. $\psi(E_p(x, y), X, Y, L_1, I_1, F_1)$ can be derived through the Fresnel diffraction integral[3]

$$\psi(E_p(x, y), X, Y, L_1, I_1, F_1) = -\frac{k_0^2\exp[\mathrm{i}k_0(L_1+I_1)]}{4\pi^2 L_1 I_1}\iint_{-\infty}^{+\infty}\exp\left[\mathrm{i}k_0\frac{(X-x_1)^2+(Y-y_1)^2}{2I_1}\right]t_{o1}(x_1,y_1)$$
$$\left\{\iint_{-\infty}^{+\infty}E_p(x,y)\exp\left[\mathrm{i}k_0\frac{(x-x_1)^2+(y-y_1)^2}{2L_1}\right]\mathrm{d}x\mathrm{d}y\right\}\mathrm{d}x_1\mathrm{d}y_1 \tag{S7}$$

If $1 / L_1 + 1 / I_1 = 1 / F_1$, Supplementary Eq. (S7) is transformed into

$$\psi(E_p(x, y), X, Y, L_1, I_1, F_1) = -\frac{L_1}{I_1}\exp\left[\frac{\mathrm{i}k_0 L_1}{F_1}\left(I_1+\frac{X^2+Y^2}{2I_1}\right)\right]E_p\left(-\frac{L_1}{I_1}X,-\frac{L_1}{I_1}Y\right) \tag{S8}$$

When the meta-differentiator is placed on the real image plane of the objective 1, I_1 and L_1 should satisfy Gaussian imaging formula $1 / L_1 + 1 / I_1 = 1 / F_1$ and $I_1 > F_1$. The electric field at the right side of the meta-differentiator is $t(X, Y)E_{SF}(X, Y)$, where $E_{SF}(X, Y)$ is derived via Supplementary Eq. (S8). At the same time, the meta-differentiator is placed at a distance of L_2 in front of the objective 2. $t(X, Y)E_{SF}(X, Y)$ and the output field, $E_{out}(x, y)$ at a distance I_2 after the objective 2, do not satisfy the Gaussian imaging

formula, i.e., $1 / L_2 + 1 / I_2 \neq 1 / F_2$. The output electric field can be transformed into $\psi(t(X, Y)E_{SF}(X, Y), x, y, L_2, I_2, F_2)$, where

$$\psi(t(X, Y)E_{SF}(X, Y), x, y, L_2, I_2, F_2) = \frac{k_0\exp[ik_0(L_2+I_2)]}{2i\pi(L_2+I_2-L_2I_2/F_2)}\exp\left[-\frac{0.5ik_0(x^2+y^2)(L_2-F_2)}{L_2F_2-I_2(L_2-F_2)}\right]$$

$$\iint_{-\infty}^{+\infty} t(X, Y)E_{SF}(X, Y)\exp\left[-\frac{0.5ik_0(X^2+Y^2)(I_2-F_2)}{I_2F_2-L_2(I_2-F_2)}\right]\exp\left(-ik_0\frac{xX+yY}{L_2+I_2-L_2I_2/F_2}\right)dXdY \quad (S9)$$

When the back focal plane of the objective 1 is the object plane of the objective 2, we have $\frac{1}{I_1-F_1+L_2}+\frac{1}{I_2}=\frac{1}{F_2}$, and Supplementary Eq. (S9) is further transformed into

$$E_{out}(x, y) = -\frac{L_1k_0\exp[ik_0(L_1+I_1+L_2+I_2)]}{2i\pi I_1(L_2+I_2-L_2I_2/F_2)}\exp\left[-\frac{0.5ik_0(x^2+y^2)(L_2-F_2)}{L_2F_2-I_2(L_2-F_2)}\right]$$

$$\iint_{-\infty}^{+\infty} t(X, Y)E_p\left(-\frac{L_1}{I_1}X,-\frac{L_1}{I_1}Y\right)\exp\left(-ik_0\frac{xX+yY}{L_2+I_2-L_2I_2/F_2}\right)dXdY \quad (S10)$$

According to Eq. (2) in the main text, the last integral term in Supplementary Eq. (S10) can be simplified into

$$\iint_{-\infty}^{+\infty} t(X, Y)E_p\left(-\frac{L_1}{I_1}X,-\frac{L_1}{I_1}Y\right)\exp\left[\frac{iL_1}{I_1}\left(k_xX+k_yY\right)\right]dXdY=$$

$$\sum_{j=1}^{l}\left[C_j\frac{(-I_1/L_1)^{2+m_j+n_j}\partial^{m_j+n_j}}{(\partial k_x)^{m_j}(\partial k_y)^{n_j}}\right]A_p\left(k_x, k_y\right) \quad (S11)$$

where $k_x = -\frac{k_0I_1x/L_1}{L_2+I_2-L_2I_2/F_2}$ and $k_y = -\frac{k_0I_1y/L_1}{L_2+I_2-L_2I_2/F_2}$.

By incorporating Supplementary Eq. (S11) into Supplementary Eq. (S10), we can get

$$E_{out}(x, y) = -\frac{I_1k_0\exp[ik_0(L_1+I_1+L_2+I_2)]}{2i\pi L_1(L_2+I_2-L_2I_2/F_2)}\exp\left[-\frac{0.5ik_0(x^2+y^2)(L_2-F_2)}{L_2F_2-I_2(L_2-F_2)}\right]\sum_{j=1}^{l}\left[C_j\frac{(-I_1/L_1)^{m_j+n_j}\partial^{m_j+n_j}}{(\partial k_x)^{m_j}(\partial k_y)^{n_j}}\right]A_p\left(k_x, k_y\right) \quad (S12)$$

The electric field amplitude of the output field can be written as $|E_{out}(x, y)| = \frac{I_1k_0}{2\pi L_1(L_2+I_2-L_2I_2/F_2)}\left|\sum_{j=1}^{l}\left[C_j\frac{(-I_1/L_1)^{m_j+n_j}\partial^{m_j+n_j}}{(\partial k_x)^{m_j}(\partial k_y)^{n_j}}\right]A_p\left(k_x, k_y\right)\right|$, which is the right differentiation of the object angular spectrum.

Supplementary Note 8: Derivation of angular spectrum differentiation for three parallel rectangular holes

The corresponding angular spectrum of the field described by Eq. (4) is $A_x(k_x, k_y) = \frac{2\sin(0.5k_xW_x)}{k_x}\frac{2\sin(0.5k_yW_y)}{k_y}[1+2\cos(2k_xW_x)]$. Under the differentiation operation $\frac{\partial^2}{\partial k_x \partial k_y}$, $\frac{\partial}{\partial k_x}$ and $\frac{\partial}{\partial k_x}+\frac{\partial}{\partial k_y}$, the angular spectrum is transformed to $S_1(k_x)S_2(k_y)$, $S_1(k_x)\frac{2\sin(0.5k_yW_y)}{k_y}$ and $S_1(k_x)\frac{2\sin(0.5k_yW_y)}{k_y}+\frac{2\sin(0.5k_xW_x)}{k_x}[1+2\cos(2k_xW_x)]S_2(k_y)$, respectively, where $S_1(k_x) = \frac{\sum_{M=1}^{3}(-1)^{M+1}(2M-1)k_xW_x\cos[(M-0.5)k_xW_x]+2(-1)^M\sin[(M-0.5)k_xW_x]}{k_x^2}$ and $S_2(k_y) = \frac{k_yW_y\cos(0.5k_yW_y)-2\sin(0.5k_yW_y)}{k_y^2}$.

Supplementary Note 9: Angular spectrum differentiation of a single circular hole

For a single circular hole, the transmitted electric field can be written as $E_x(x,y)=\begin{cases}1 & \left(\sqrt{x^2+y^2}\le R\right)\\ 0 & \left(\sqrt{x^2+y^2}>R\right)\end{cases}$, with the angular spectrum of $A_x(k_x, k_y) = 2\pi R\frac{J_1\left(R\sqrt{k_x^2+k_y^2}\right)}{\sqrt{k_x^2+k_y^2}}$. With $\frac{\partial^2}{\partial k_x \partial k_y}$, $\frac{\partial}{\partial k_x}$ and $\frac{\partial}{\partial k_x}+\frac{\partial}{\partial k_y}$, the angular spectrum is transformed to $\frac{2\pi k_x k_y}{\sqrt{k_x^2+k_y^2}}\frac{\left[8\text{-}R^2\left(k_x^2+k_y^2\right)\right]J_1\left(R\sqrt{k_x^2+k_y^2}\right)\text{-}4R\sqrt{k_x^2+k_y^2}J_0\left(R\sqrt{k_x^2+k_y^2}\right)}{\left(k_x^2+k_y^2\right)^2}$, $\frac{2\pi R k_x}{\sqrt{k_x^2+k_y^2}}\frac{R\sqrt{k_x^2+k_y^2}J_0\left(R\sqrt{k_x^2+k_y^2}\right)\text{-}2J_1\left(R\sqrt{k_x^2+k_y^2}\right)}{k_x^2+k_y^2}$ and $\frac{2\pi R\left(k_x+k_y\right)}{\sqrt{k_x^2+k_y^2}}\frac{R\sqrt{k_x^2+k_y^2}J_0\left(R\sqrt{k_x^2+k_y^2}\right)\text{-}2J_1\left(R\sqrt{k_x^2+k_y^2}\right)}{k_x^2+k_y^2}$, respectively, where J_0 and J_1 are the zeroth order and the first order Bessel functions of the first kind, respectively. The transmitted electric field after the object is plotted in the first panel of Supplementary Fig. S10a. The associated normalized angular spectrum $|A_x(k_x, k_y)|^2$ is plotted in the first panel of Supplementary Fig. S10b, with the intensity profile being shown in the first panel of Supplementary Fig. S10c. The theoretical angular spectrum differentiations for $\frac{\partial^2}{\partial k_x \partial k_y}$, $\frac{\partial}{\partial k_x}$ and $\frac{\partial}{\partial k_x}+\frac{\partial}{\partial k_y}$ are shown in the first panel of Supplementary Figs. S10d, f, h, respectively, where the associated intensity profiles along lines A-B are plotted in the first panel of Supplementary Figs. S10e, g, i, respectively. The angular spectrum at 685.5, 532 and 450 nm are experimentally extracted and recorded by the CCD camera, as shown in the second to the fourth panels in Supplementary Figs. S10a-i. The experiment is well consistent with the above theoretical results. It can be seen that, under the angular spectrum differentiation of $\frac{\partial^2}{\partial k_x \partial k_y}$, the central spot in the angular spectrum is transformed into four spots (Supplementary Figs. S10d, e). The central spot is only transformed into two spots with $\frac{\partial}{\partial k_x}$ in Supplementary Figs. S10f, g. The angular spectrum experiences a first-order differentiation along $\mathbf{k_x} + \mathbf{k_y}$ direction with $\frac{\partial}{\partial k_x}+\frac{\partial}{\partial k_y}$, as $\frac{\partial}{\partial k_x}+\frac{\partial}{\partial k_y}$ is equivalent to $\frac{2\partial}{\partial\left(k_x+k_y\right)}$. As a result, the central spot is transformed into two spots along $\mathbf{k_x} + \mathbf{k_y}$ direction (Supplementary Figs. S10h, i).

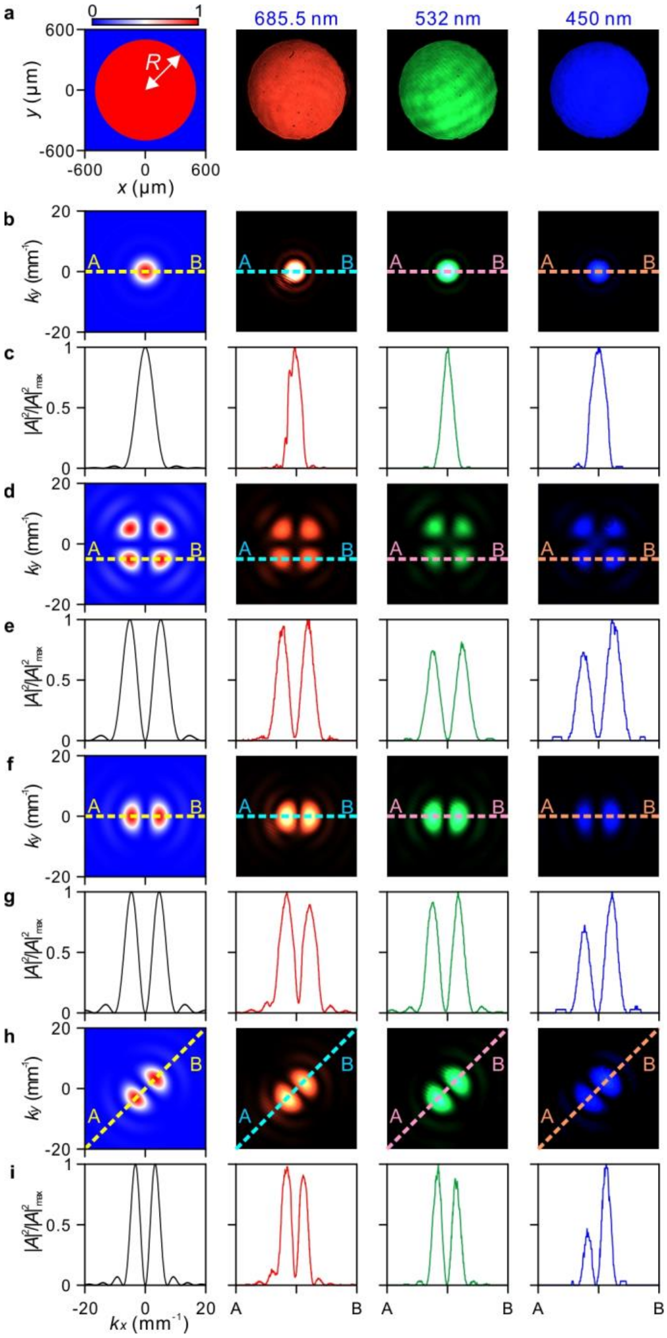

Supplementary Figure S10. Measured angular spectrum differentiations of a single circular hole. **a** Theoretical (the first panel) and experimental (the second to fourth panels) intensity distributions of a single circular hole drilled in a stainless steel plate (1 mm thick). The hole has a radius of R = 500 μm. **b, c** Angular spectrum intensity distributions (**b**) and normalized intensity profiles along lines A-B in (**b**) (**c**). **d-i** Angular spectrum intensity distributions with $\frac{\partial^2}{\partial k_x \partial k_y}$ (**d**), $\frac{\partial}{\partial k_x}$ (**f**), and $\frac{\partial}{\partial k_x}+\frac{\partial}{\partial k_y}$ (**h**), and their normalized intensity profiles along lines A-B (**e, g, i**).

Supplementary Note 10: Derivation of transmitted electric field when a meta-differentiator is involved in a 4F system

Edge detection is commonly explored to characterize subject boundaries of an image, and is a powerful tool of imaging processing for object detection[4-9]. Previously, optical metamaterials and metasurfaces have been suggested to perform edge detection using spatial differentiation, but are largely restricted to narrow operating bandwidth[5,8,10-13]. This may severely limit the capability of exploiting wavelength division multiplexing to enhance data processing speed. In contrast, the angular spectrum meta-differentiators have the merit of implementing angular spectrum differentiation, regardless of operating wavelength. The conventional diffractive optical elements can be combined with 4F systems for angular spectrum processing, leading to various imaging applications, such as dark-field[14,15], schlieren[16,17], and phase-contrast imaging[18].

The meta-differentiators can be combined with a confocal system consisting of two objectives to demonstrate the broadband and versatile edge-detection imaging capabilities (Supplementary Fig. S11a). The process can be obtained by setting $I_1 = F_1$ and $L_2 = F_2$ in Supplementary Fig. S9. Besides, the relationship $\frac{L_1 F_1}{L_1\text{-}F_1}+\frac{I_2 F_2}{I_2\text{-}F_2}=F_1+F_2$ should be satisfied for the system to image the input field without the meta-differentiator. The field $E_{SF}(X, Y) = \psi(E_p(x, y), X, Y, L_1, F_1, F_1)$ and the output field is $E_{out}(x, y) = \psi(t(X, Y)E_{SF}(X, Y), x, y, F_2, I_2, F_2)$. Combing them with Supplementary Eq. (S9), the output field is $E_{out}(x, y) = -\frac{F_1 \exp[\mathrm{i}k_0(L_1+F_1+F_2+I_2)]}{F_2}\sum_{j=1}^{l} C_j \left(\frac{F_2}{k_0}\right)^{m_j+n_j} \frac{\partial^{m_j+n_j}}{(\partial x)^{m_j}(\partial y)^{n_j}} E_p\left(-\frac{F_1}{F_2}x, -\frac{F_1}{F_2}y\right)$. Neglecting the phase term $\exp[\mathrm{i}k_0(L_1 + F_1 + F_2 + I_2)]$,

$$E_{out}(x, y) = -\frac{F_1}{F_2}\sum_{j=1}^{l} C_j \left(\frac{F_2}{k_0}\right)^{m_j+n_j} \frac{\partial^{m_j+n_j}}{(\partial x)^{m_j}(\partial y)^{n_j}} E_p\left(-\frac{F_1}{F_2}x, -\frac{F_1}{F_2}y\right) \tag{S13}$$

This equation indicates that, the transmitted waveform is the differentiation of the input electric field, arising from the meta-differentiator modulation. In addition, the transmitted electric field is scaled and inverse with regard to the input electric field, where the scale ratio is determined by F_1/F_2. Specifically, the optical systems comprised of the aforementioned meta-differentiators and two objectives can provide three types of spatial differentiation as $-C_1\frac{F_1 F_2}{k_0^2}\frac{\partial^2}{\partial x \partial y}E_p\left(-\frac{F_1}{F_2}x, -\frac{F_1}{F_2}y\right)$, $-C_1\frac{F_1}{k_0}\frac{\partial}{\partial x}E_p\left(-\frac{F_1}{F_2}x, -\frac{F_1}{F_2}y\right)$ and $-C_1\frac{F_1}{k_0}\left(\frac{\partial}{\partial x}+\frac{\partial}{\partial y}\right)E_p\left(-\frac{F_1}{F_2}x, -\frac{F_1}{F_2}y\right)$, respectively and the corresponding transmitted electric field $E_{out}(x, y)$ is listed in Supplementary Table S3. It can be inferred by comparing

Supplementary Eq. (S13) and Eq. (2) that, the resultant transmitted electric field $E_{out}(x, y)$ distributions in the spatial domain resemble those in the angular spectrum domain, when the input electric field passes through the corresponding meta-differentiator only.

Supplementary Table S3 | l, C_j and (m_j, n_j) for the three types of meta-differentiators

l	$l = 1$		$l = 2$
C_j	C_1	C_1	$C_1 = C_2$
(m_j, n_j)	$(m_1, n_1) = (1, 1)$	$(m_1, n_1) = (1, 0)$	$(m_1, n_1) = (1, 0)$,<br>$(m_2, n_2) = (0, 1)$
$t(X, Y)$	$-C_1XY$	$-\mathrm{i}C_1X$	$-\mathrm{i}C_1(X + Y)$
$E_{out}(x, y)$	$-C_1\frac{F_1F_2}{k_0^2}\frac{\partial^2}{\partial x\partial y}$ $E_p\left(-\frac{F_1}{F_2}x, -\frac{F_1}{F_2}y\right)$	$-C_1\frac{F_1}{k_0}\frac{\partial}{\partial x}$ $E_p\left(-\frac{F_1}{F_2}x, -\frac{F_1}{F_2}y\right)$	$-C_1\frac{F_1}{k_0}\left(\frac{\partial}{\partial x}+\frac{\partial}{\partial y}\right)$ $E_p\left(-\frac{F_1}{F_2}x, -\frac{F_1}{F_2}y\right)$

To experimentally show how the differentiation type affects the edge-detection imaging, we used the meta-differentiators to enable edge-detection imaging of an object (Supplementary Fig. S11a). The 1951 United State Air Force (USAF) resolution test chart has a high transmission contrast between opaque and transparent regions. The recorded images with a negative resolution test chart are shown with red, green and blue illumination (Supplementary Figs. S11b-d). Due to the absence of the meta-differentiators, the transparent regions on the test chart, such as rectangular holes, correspond to bright area on the image due to high transmission, while the opaque regions lead to dark areas on the image due to zero transmission (see the first column in Supplementary Figs. S11b-d). It is interesting to find that, different differentiation types lead to different edge-detection images. For the second column with $\hat{H} = C_1\frac{\partial^2}{\partial k_x\partial k_y}$, the four edges of the rectangle along x and y directions become dark, and the remaining vertices are bright only. For the third column with $\hat{H} = C_l\frac{\partial}{\partial k_x}$, the two edges of the rectangle along x direction become dark, while the vertical edges along y direction are kept bright. For the fourth column with $\hat{H} =$

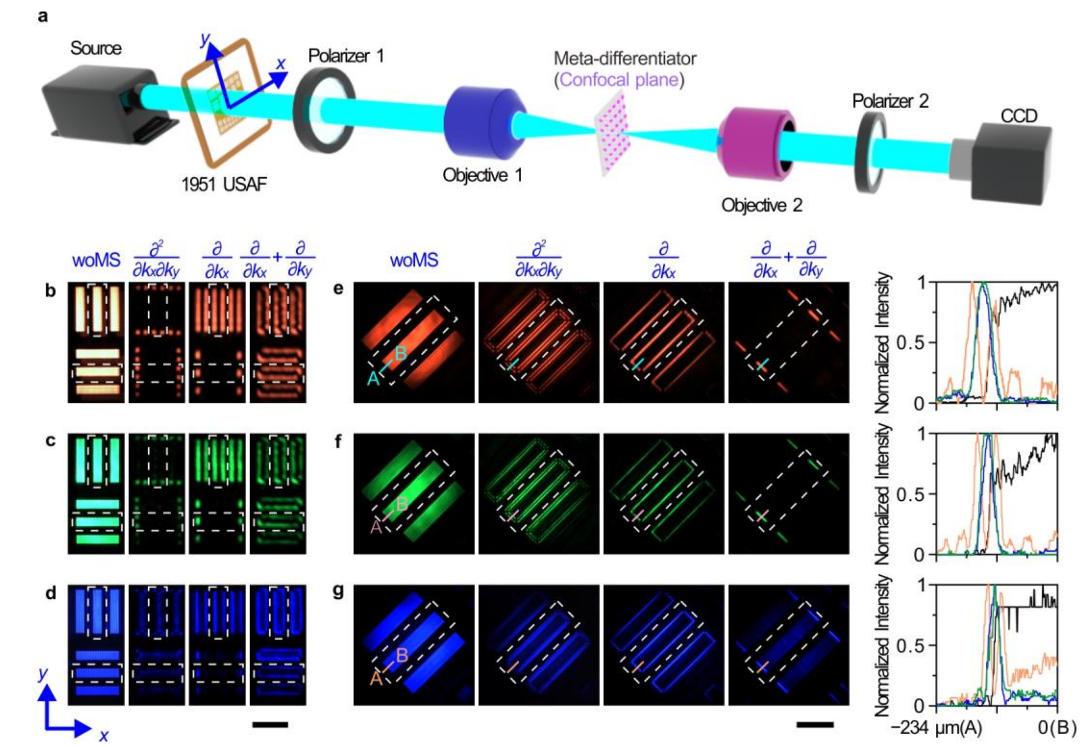

Supplementary Figure S11. Edge detection of the negative 1951 USAF resolution test chart. a Experimental setup for edge-detection imaging. The meta-differentiator is on the confocal plane of the two objectives. **b-g** Field intensity distributions without the metasurfaces and with the metasurfaces realizing $\frac{\partial^2}{\partial k_x \partial k_y}$, $\frac{\partial}{\partial k_x}$ and $\frac{\partial}{\partial k_x}+\frac{\partial}{\partial k_y}$, respectively, when the 1951 USAF resolution test chart was illuminated under red (**b, e**), green (**c, f**), and blue light (**d, g**). The test chart was rotated 45° clockwise in (**e-g**) with respect to that in (**b-d**). In the last column, the black, yellow, blue and green curves show the normalized field intensity profiles along lines A-B in (**e-g**). The scale bars are 300 μm and 500 μm in (**b-d**) and (**e-g**), respectively.

$C_1(\frac{\partial}{\partial k_x}+\frac{\partial}{\partial k_y}) = 2C_1\frac{\partial}{\partial (k_x+k_y)}$, such process undergoes a first-order partial derivative with respect to $x + y$, the left-upper and right-lower vertices of the rectangle become dark only. To clearly present the difference between the second-order and the first-order differentiations, we further rotated the resolution test chart with 45° clockwise (Supplementary Figs. S11e-g) for edge-detection imaging. In this case, the areas associated with the rectangles are still bright without the meta-differentiators (see the first column). For the second column in Supplementary Figs. S11e-g, the four vertices of a rectangle become dark, and each edge splits into two parallel bright lines. The reason that causes edge splitting can be explained as follows. In this case, $\frac{\partial^2}{\partial x \partial y}$ equals $\frac{\partial^2}{\partial (x+y)^2}-\frac{\partial^2}{\partial (x-y)^2}$. The first and second items $\frac{\partial^2}{\partial (x+y)^2}$ and $-\frac{\partial^2}{\partial (x-y)^2}$ result in

edge along **x** − **y** and **x** + **y** directions splitting, respectively. For the third column, the two vertices of a rectangle at the top and bottom become dark only. For the fourth column with $\frac{\partial}{\partial x}+\frac{\partial}{\partial y} = \frac{2\partial}{\partial(x+y)}$, the two edges along **x** + **y** direction become dark and the other two edges along **x** − **y** direction remain bright. The normalized intensity profile along the dashed lines A-B in Supplementary Figs. S11e-g is extracted and plotted in the fifth column, clearly indicating that the intensity at the opaque/transparent interface has a rising step. The intensity profile at the interface splits into one peak through the two types of first-order differentiation, but branches into two peaks for second-order differentiation.

In addition, we have explored the realization of the partial derivative using polar coordinates. Let us take $E_{out}(x, y) = \frac{\partial E_p(x, y)}{\partial\sqrt{x^2+y^2}} = \frac{\partial E_p(x, y)}{\partial r}$ as an example, and derive the required real-space transfer function, $t(X, Y)$, that should be realized by the meta-differentiator. To simplify the mathematical derivation, it is assumed that $F_1 = F_2 = F$ in the 4F system (Supplementary Fig. S11). Based on the theoretical model in Supplementary Note 7, the output field can be expressed as $E_{out}(x, y) = -\frac{k_0^2\exp(\mathrm{i}4k_0F)}{4\pi^2F^2}\iint_{-\infty}^{+\infty} t(X,Y)A_p\left(\frac{k_0X}{F},\frac{k_0Y}{F}\right)\exp\left(-\mathrm{i}\frac{k_0x}{F}X-\mathrm{i}\frac{k_0y}{F}Y\right)\mathrm{d}X\mathrm{d}Y$ in Cartesian coordinates. We can thus derive the required real-space transfer function as $t(X, Y) = -\exp(-\mathrm{i}4k_0F)\frac{\iint_{-\infty}^{+\infty}\frac{\partial E_p(x, y)}{\partial\sqrt{x^2+y^2}}\exp\left(\mathrm{i}\frac{k_0X}{F}x+\mathrm{i}\frac{k_0Y}{F}y\right)\mathrm{d}x\mathrm{d}y}{A_p\left(\frac{k_0X}{F},\frac{k_0Y}{F}\right)}$. $t(X, Y)$ can be simplified as $t(X, Y) = \frac{k_0\exp(-\mathrm{i}4k_0F)}{2\pi F}\iint_{-\infty}^{+\infty}\frac{(Xx+x^2+Yy+y^2)A_p\left(\frac{k_0x}{F},\frac{k_0y}{F}\right)}{A_p\left(\frac{k_0X}{F},\frac{k_0Y}{F}\right)\left[(X+x)^2+(Y+y)^2\right]^{1.5}}\mathrm{d}x\mathrm{d}y$. $t(X, Y)$ is equivalent to $t(R, \phi) = \frac{k_0\exp(-\mathrm{i}4k_0F)}{2\pi F}\int_0^{2\pi}\int_0^{+\infty}\frac{\left[r^3+Rr^2\cos(\varphi-\phi)\right]A_p\left(\frac{k_0r}{F}\cos\varphi,\frac{k_0r}{F}\sin\varphi\right)}{A_p\left(\frac{k_0R}{F}\cos\phi,\frac{k_0R}{F}\sin\phi\right)\left[R^2+r^2+2Rr\cos(\varphi-\phi)\right]^{1.5}}\mathrm{d}r\mathrm{d}\varphi$ in polar coordinates, where $r = \sqrt{x^2+y^2}$, $x = r\cos\varphi$, $y = r\sin\varphi$, $R = \sqrt{X^2+Y^2}$, $X = R\cos\phi$, $Y = R\sin\phi$. $t(R, \phi)$ is not dependent on R and ϕ but also r and φ, and hence the required $t(R, \phi)$ is varied with the incident light. As a result, it might be unable to construct the partial derivative, $\frac{\partial}{\partial r}$, so as to realize edge-detection imaging, regardless of directions.

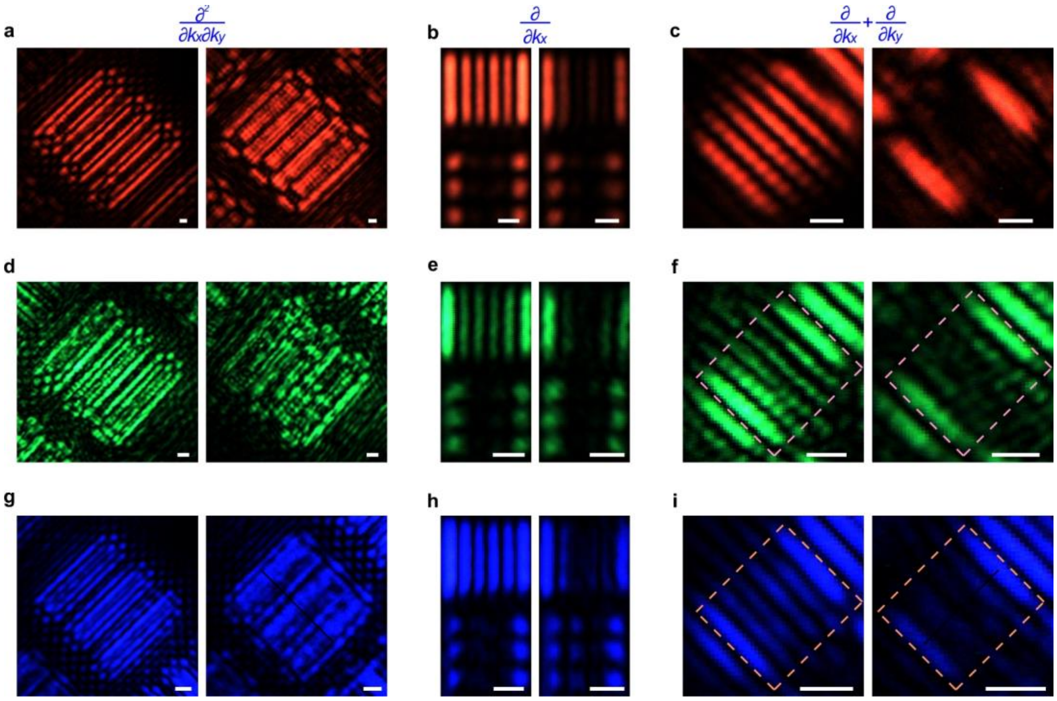

Supplementary Figure S12. Resolutions test. a-i Field intensity distributions with the meta-differentiators realizing $\frac{\partial^2}{\partial k_x \partial k_y}$ (**a, d, g**), $\frac{\partial}{\partial k_x}$ (**b, e, h**) and $\frac{\partial}{\partial k_x}+\frac{\partial}{\partial k_y}$ (**c, f, i**), respectively, when a negative resolution test chart was illuminated under red (**a-c**), green (**d-f**), and blue light (**h-i**). The regions bounded by the dashed boxes are associated with three parallel rectangular holes. All the scale bars are 50 μm.

The negative resolution test chart is also used to evaluate the smallest linewidths that the meta-differentiators can resolve. In Supplementary Figs. S12a, d, g with $\hat{H} = C_1 \frac{\partial^2}{\partial k_x \partial k_y}$ and Supplementary Figs. S12c, f, i with $\hat{H} = C_1 (\frac{\partial}{\partial k_x}+\frac{\partial}{\partial k_y})$, the resolution test chart is rotated with 45° clockwise with respect to those in Supplementary Figs. S12b, e, h. The left figures in Supplementary Figs. S12a-i represent the smallest linewidths that the meta-differentiator can distinguish with different differentiation types and at different wavelengths. When the linewidths are further reduced, they are undistinguishable with the meta-differentiator, which is shown in the right figures in Supplementary Figs. S12a-i. The resolution values with different differentiation types and at different wavelengths are summarized in Supplementary Table S4.

Supplementary Table S4 | Resolutions for the meta-differentiators

wavelength \ resolution \ $\hat{H}$	$\frac{\partial^2}{\partial k_x \partial k_y}$	$\frac{\partial}{\partial k_x}$	$\frac{\partial}{\partial k_x}+\frac{\partial}{\partial k_y}$
685.5 nm	111.36 μm	39.37 μm	35.08 μm
532 nm	78.75 μm	24.80 μm	24.80 μm
450 nm	62.50 μm	24.80 μm	19.69 μm

The edge-detection imaging based on a positive resolution test chart for the three differentiation types are also experimentally demonstrated (Supplementary Fig. S13).

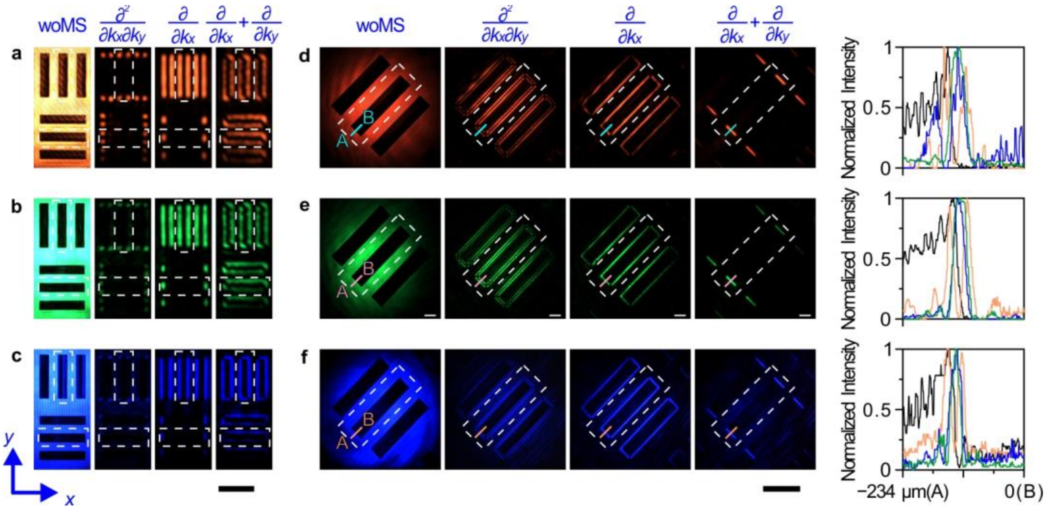

Supplementary Figure S13. Edge detection of the positive 1951 USAF resolution test chart. a-f Field intensity distributions without the metasurfaces and with metasurfaces realizing $\frac{\partial^2}{\partial k_x \partial k_y}$, $\frac{\partial}{\partial k_x}$ and $\frac{\partial}{\partial k_x}+\frac{\partial}{\partial k_y}$, respectively when a positive resolution test chart was illuminated under red (**a, d**), green (**b, e**), and blue light (**c, f**). The test chart was rotated 45° clockwise in (**d-f**) with respect to that in (**a-c**). In the last column, the black, yellow, blue and green curves show the normalized field intensity profiles along lines A-B in (**d-f**). The scale bars are 300 μm and 500 μm in (**a-c**) and (**d-f**), respectively.

Optical imaging of biological cells is difficult to observe the small details of biological samples due to the smooth refractive index difference across biological cells[9,19]. A biological cell has much worse transmission contrast and more complex texture, as opposed to a test chart. Direct imaging of a frog egg cell without meta-differentiators leads to a vague image, and one even cannot distinguish the cell membrane (a thick rigid structure) and the interior cell cytoplasm (the first column of Supplementary Fig.

S14). The small details in the cell cytoplasm are undistinguishable with illumination of red, green and blue colors. At the presence of meta-differentiators, the image contrast is completely changed. Not only the edge imaging on the cell membrane is enhanced and clearly distinguished (second to forth columns of Supplementary Fig. S14), but also the interior cell cytoplasm that was observed to be completely vague in the first column of Supplementary Fig. S14 becomes distinguishable (see the regions enclosed with white dashed lines in the third to forth columns of Supplementary Fig. S14). Meanwhile, we observed the edge splitting, where some membrane boundaries are bright and other remaining boundaries are dark, determined by the differentiation type.

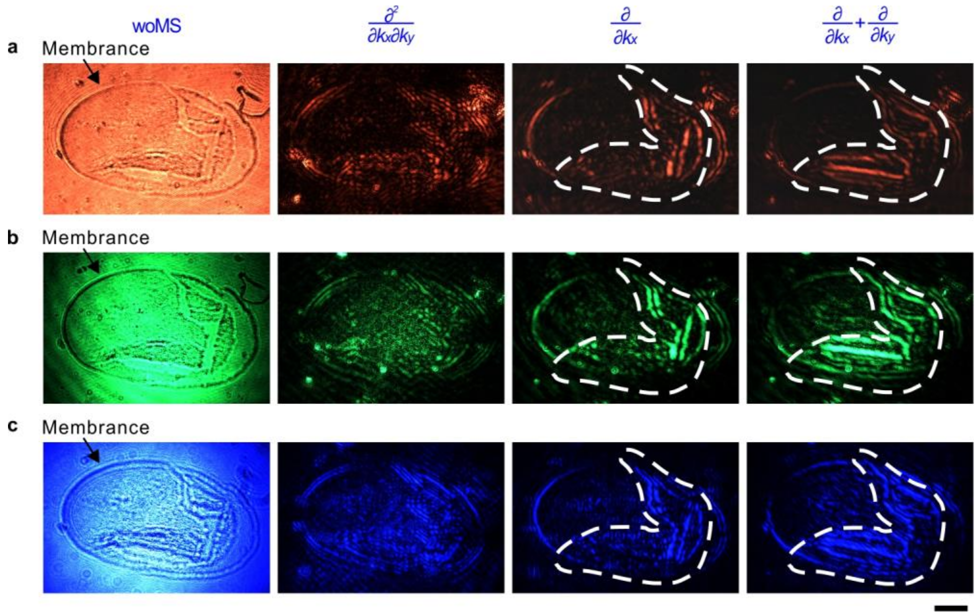

Supplementary Figure S14. Edge detection of a frog egg cell. a-c Field intensity distributions without the metasurfaces and with the metasurfaces realizing $\frac{\partial^2}{\partial k_x \partial k_y}$, $\frac{\partial}{\partial k_x}$ and $\frac{\partial}{\partial k_x}+\frac{\partial}{\partial k_y}$, respectively, when the frog egg cell was illuminated under red (**a**), green (**b**), and blue light (**c**). The scale bar is 200 μm.

Supplementary Note 11: Transmission efficiency of the cross-polarized transmitted field

Under the x-polarized incidence, the y-polarized transmitted light is used to perform the differentiation operation. We have simulated and measured the transmission efficiency of the transmitted y-polarized component. In the simulation, η_{peak} and η_{int} can be evaluated by $\eta_{peak} = \frac{4n_s}{(1+n_s)^2}\frac{\max\left[|A_y(k_x,k_y)|^2\right]}{n_s\max\left[|A_x(k_x,k_y)|^2\right]}$ and $\eta_{int} = \frac{4n_s}{(1+n_s)^2}\frac{\iint_{-\infty}^{+\infty}|A_y(k_x,k_y)|^2\mathrm{d}k_x\mathrm{d}k_y}{n_s\iint_{-\infty}^{+\infty}|A_x(k_x,k_y)|^2\mathrm{d}k_x\mathrm{d}k_y}$, respectively, where n_S is the refractive index of the silica substrate. $A_x(k_x, k_y)$ and $A_y(k_x, k_y)$ are the angular spectrum of the input x-polarized electric field $E_x(x, y)$ and the transmitted y-polarized electric field $E_y(x, y)$, respectively. The transmission efficiencies for the three types of meta-differentiators at three wavelengths used in Fig. 2 and Supplementary Fig. S3 are listed in Supplementary Table S5.

Supplementary Table S5 | η_{peak} and η_{int} for the three types of meta-differentiators used in Fig. 2 and Supplementary Fig. S3

$\hat{H}$ / wavelength η_{peak}/η_{int}	$\frac{\partial^2}{\partial k_x \partial k_y}$	$\frac{\partial}{\partial k_x}$	$\frac{\partial}{\partial k_x}+\frac{\partial}{\partial k_y}$
685.5 nm	$1.9\times10^{-4}/3.5\times10^{-4}$	$5.6\times10^{-3}/7.6\times10^{-3}$	$2.8\times10^{-3}/3.9\times10^{-3}$
532 nm	$3.2\times10^{-5}/9.0\times10^{-5}$	$9.0\times10^{-4}/1.2\times10^{-3}$	$4.4\times10^{-4}/5.9\times10^{-4}$
450 nm	$3.1\times10^{-5}/1.0\times10^{-4}$	$8.8\times10^{-4}/1.2\times10^{-3}$	$4.2\times10^{-4}/5.7\times10^{-4}$

In the experiment, the x-polarized transmitted light is removed by polarizer 2, and hence the light field recorded by CCD is dominated by y-polarized light, associated with metasurface differentiation operation. The transmission efficiencies for the three types of meta-differentiators at three wavelengths used in Fig. 4 are listed in Supplementary Table S6.

Supplementary Table S6 | η_{peak} and η_{int} for the three types of meta-differentiators used in Fig. 4

$\hat{H}$ / wavelength η_{peak}/η_{int}	$\frac{\partial^2}{\partial k_x \partial k_y}$	$\frac{\partial}{\partial k_x}$	$\frac{\partial}{\partial k_x}+\frac{\partial}{\partial k_y}$
685.5 nm	$2.8\times10^{-4}/5.1\times10^{-4}$	$4.8\times10^{-3}/4.8\times10^{-3}$	$1.8\times10^{-3}/2.3\times10^{-3}$
532 nm	$1.8\times10^{-5}/2.7\times10^{-5}$	$3.2\times10^{-4}/2.3\times10^{-4}$	$9.6\times10^{-5}/8.5\times10^{-5}$
450 nm	$0.9\times10^{-5}/1.4\times10^{-5}$	$7.8\times10^{-5}/7.1\times10^{-5}$	$4.9\times10^{-5}/3.8\times10^{-5}$

According to Eq. (3) in the main text, the local transmission efficiency for y-polarized component is dependent by $\left|\frac{t_u - t_v}{2}\right|$. It can be inferred that, a feasible method of enhancing the transmission efficiency can be realized by using high-aspect-ratio dielectric nanopillars, made by low-loss materials, such as crystalline silicon and TiO_2. Here, we have employed high-aspect-ratio crystalline silicon as the dielectric nanopillar for designing the meta-differentiators, where crystalline silicon has lower loss, as opposed to amorphous silicon deposited by magnetron sputtering in our experiment. The crystalline silicon metasurface on a silica substrate has a thickness of 600 nm, with W_u = 140 nm, W_v = 60 nm and P = 280 nm. The crystalline silicon nanopillar has a larger value of $\left|\frac{t_u - t_v}{2}\right|$ than that of the nanopillar by amorphous silicon (Supplementary Fig. S6e). The transmission efficiencies for the three types of meta-differentiators (60×60 nanopillars) at three wavelengths are listed in Supplementary Table S7. The transmission efficiencies are increased by approximately 3-21 fold.

Supplementary Table S7 | η_{peak} and η_{int} for the three types of meta-differentiators with crystalline silicon nanopillars

$\hat{H}$ / wavelength \ η_{peak}/η_{int}	$\frac{\partial^2}{\partial k_x \partial k_y}$	$\frac{\partial}{\partial k_x}$	$\frac{\partial}{\partial k_x}+\frac{\partial}{\partial k_y}$
685.5 nm	$5.7\times10^{-4}/1.0\times10^{-3}$	$1.8\times10^{-2}/2.4\times10^{-2}$	$8.9\times10^{-3}/1.2\times10^{-2}$
532 nm	$6.4\times10^{-4}/1.1\times10^{-3}$	$1.8\times10^{-2}/2.5\times10^{-2}$	$9.1\times10^{-3}/1.2\times10^{-2}$
450 nm	$3.0\times10^{-4}/7.8\times10^{-4}$	$6.6\times10^{-3}/9.2\times10^{-3}$	$3.6\times10^{-3}/5.1\times10^{-3}$

Supplementary References

1. Guo, W.-L. *et al.* Airy beam generation: approaching ideal efficiency and ultra wideband with reflective and transmissive metasurfaces. *Adv. Opt. Mater.* **8**, 2000860 (2020).
2. Zhou, N. *et al.* Ultra-compact broadband polarization diversity orbital angular momentum generator with 3.6 × 3.6 μm^2 footprint. *Sci. Adv.* **5**, eaau9593 (2019).
3. Cubillos, M. & Jimenez, E. Diffraction integral computation using sinc approximation. *Appl. Numer. Math.* **178**, 69-83 (2022).
4. Zhou, J. *et al.* Optical edge detection based on high-efficiency dielectric metasurface. *Proc. Natl. Acad. Sci. USA* **116**, 11137-11140 (2019).

5. Pan, D. *et al.* Laplace metasurfaces for optical analog computing based on quasi-bound states in the continuum. *Photon. Res.* **9**, 1758-1766 (2021).

6. Guo, C., Xiao, M., Minkov, M., Shi, Y. & Fan, S. Photonic crystal slab Laplace operator for image differentiation. *Optica* **5**, 251-256 (2018).

7. Zhu, T. *et al.* Plasmonic computing of spatial differentiation. *Nat. Commun.* **8**, 15391 (2017).

8. Dong, Z., Si, J., Yu, X. & Deng, X. Optical spatial differentiator based on subwavelength high-contrast gratings. *Appl. Phys. Lett.* **112**, 181102 (2018).

9. Huo, P. *et al.* Photonic spin-multiplexing metasurface for switchable spiral phase contrast imaging. *Nano Lett.* **20**, 2791-2798 (2020).

10. Cordaro A. *et al.* High-index dielectric metasurfaces performing mathematical operations. *Nano Lett.* **19**, 8418-8423 (2019).

11. Wang, Z. *et al.* Single-layer spatial analog meta-processor for imaging processing. *Nat. Commun.* **13**, 2188 (2022).

12. Zhou, Y. *et al.* Analog optical spatial differentiators based on dielectric metasurfaces. *Adv. Opt. Mater.* **8**, 1901523 (2019).

13. Bao, L., Wu, R. Y., Fu, X. & Cui, T. J. Mathematical operations of transmissive near fields controlled by metasurface with phase and amplitude modulations. *Ann. Phys.* **532**, 2000069 (2020).

14. Braslavsky, I. *et al.* Objective-type dark-field illumination for scattering from microbeads. *Appl. Optics* **40**, 5650-5657 (2001).

15. Ueno, H. *et al.* Simple dark-field microscopy with nanometer spatial precision and microsecond temporal resolution. *Biophys. J.* **98**, 2014-2023 (2010).

16. Settles, G. S. & Hargather, M. J. A review of recent developments in schlieren and shadowgraph techniques. *Meas. Sci. Technol.* **28**, 042001 (2017).

17. Wang, W., Dong, R. & Yang, W. Sensitivity investigation of schlieren imaging system. *Laser Optoelectron. Prog.* **55**, 111102 (2018).

18. Zernike, F. How I discovered phase contrast. *Science* **121**, 345-349 (1955).

19. Zhou, Y., Zheng, H. Y., Kravchenko, I. I. & Valentine, J. Flat optics for image differentiation. *Nat. Photonics* **14**, 316-323 (2020).
